# Enhancement of intra-cardiac flow-field data using adaptive Kernel filtering

**DOI:** 10.1038/s41598-023-47053-4

**Published:** 2023-12-13

**Authors:** Shataneek Banerjee, Amardip Ghosh, Prasanta Pal

**Affiliations:** 1 Department of Aerospace Engineering, IIT Kharagpur, Kharagpur, India; 2 SHIOM LLC, Rhode Island Startup Incubator (RIHUB), Providence, RI USA

**Keywords:** Applied mathematics, Computational science, Computer science, Scientific data, Biomedical engineering, Mechanical engineering, Anatomy, Biomarkers, Cardiology, Medical research, Mathematics and computing

## Abstract

A method of determining the optimal kernel size for filtering noise in vortex dominated flow-fields, as found in the cardiac chambers is presented in this paper. Using synthetic flow fields generated using harmonic functions and perturbed using Gaussian noises of different amplitudes and spreads, the effect of kernel size on noise removal using the Median filter is tested systematically. It is shown that there exists an optimal kernel size at which the Median filter works best. The size of the optimal kernel is shown to be related to the vortex size. When applied to MRI generated cardiac flow-fields, the approach is seen to reveal underlying vortex patterns thereby aiding as an effective tool in the diagnosis and prognosis of cardiac diseases based on vortices as clinical biomarkers. The behavior of the restored cardiac flow fields which are filtered with the optimal kernel size and also with some values preceding and succeeding it are similar to that observed in studies with synthetic flow fields. This confirms that the optimal size of the kernel is related to the cardiac vortex size as is observed in the case of synthetic flow fields.

## Introduction

**Figure 1:**
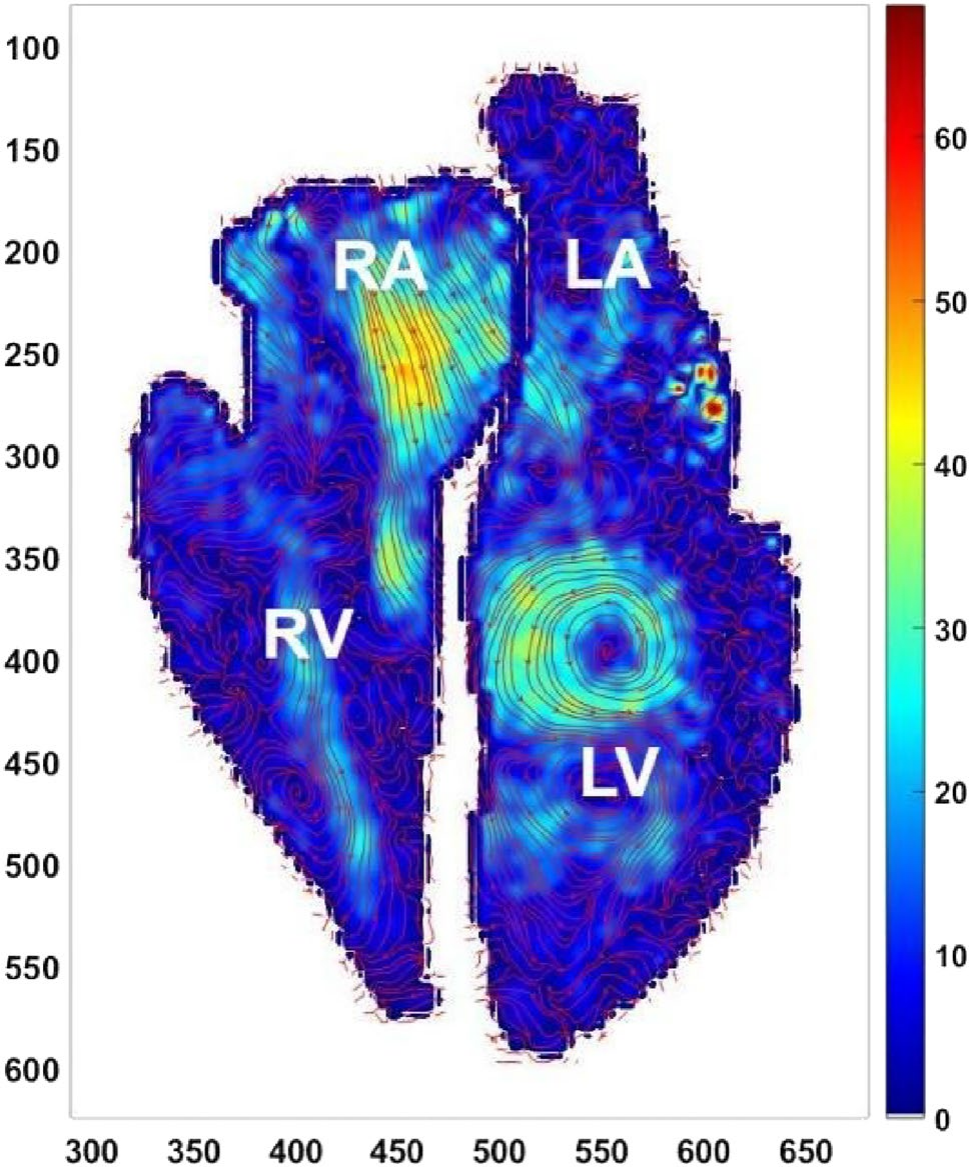
Unfiltered cardiac flow field. X and Y axes are spatial coordinates having units of pixels. Unit of colorbar, i.e., velocity is cm/s. RA represents the location of the right atrium, RV represents the location of the right ventricle, LA represents the location of the left atrium, LV represents the location of the left ventricle.

Vortices in the cardiac chambers, as shown in Fig. 1, can provide valuable information about the condition of the heart^1^. Pathological hearts are characterized by malformed and maladaptive vortices. Vortices become progressively incoherent in left ventricular remodeling which progresses into stiffening of the left ventricle leading to dilated cardiomyopathy^2^. Life span of cardiac vortices, i.e. fraction of the duration of the cardiac cycle for which the vortex is existing, serves as a biomarker for borderline mPAP and pulmonary hypertension^3,4^. Left ventricular vortex formation time which is inherently a measure of the length to diameter ratio of the ejected fluid column from the left atrium serves as an indicator for dilated cardiomyopathy^5^ and also for assessing the severity of left ventricular diastolic dysfunction^6^.

Left atrial vortex size is a biomarker for paroxysmal atrial fibrillation. PAF patients have large left atrial vortex size and it is associated with CHA2DS2-VASc risk score^7^. Left ventricular vortex length and vortex depth serves as a biomarker for left ventricular systolic dysfunction. Patients with abnormal left ventricular systolic function present lower values of left ventricular vortex length and depth values^8^. Sphericity index of left ventricular vortex, which is the ratio of vortex length to vortex width, is lower in left ventricular systolic dysfunction cases^8^. Relative strength of left ventricular vortex which represents the strength of the pulsatile vorticity with respect to the averaged vorticity in the whole left ventricle is much lower in systolic dysfunction patients than normal persons^8^. Vortical kinetic energy, i.e., the kinetic energy summed up over all the pixels inside a delineated vortex within the left ventricle is a biomarker for heart failure (NYHA, class I-IV). A smaller percentage of diastolic kinetic energy is contained within vortices in heart failure patients compared to healthy controls^9^. The effect on the cardiac flow dynamics of using prosthetic devices like mechanical prosthetic mitral valves can be assessed by the interaction between the coherent structures and the fraction of the time for which they are existing in the diastolic phase^10^. Dynamics of vortical structures from their production, diffusion, dissipation and finally to degeneration provides a good insight into the effect of myocardial wall interactions on the flow. If the frequency of the pulsatile wall interactions is decreased, degeneration of vortical structures occurs^11^. Vorticity which is actually a measure of the rate of rotation when computed in a spatially integrated manner by placing a kernel of a certain size around a point serves as a biomarker for Right Ventricular Diastolic Dysfunction^12^ and also for the severity of aortic dilatation^13^.

Hemodynamic data, obtained from Phase Contrast MRI (PC MRI) is corrupt with noise arising from various sources. Mismatch between the encoding velocity used and the local peak velocity in the region of interest affects the sensitivity of the acquisition^14,15^. Too high encoding velocity compared to the local peak velocity leads to very low sensitivity of the encoding device which makes the encoded velocity values cluttered^16^ In regions of the cardiovascular system where the flow rate is high, only the component of the noise aligned to the direction of the flow affects the flow data and the noise is correlated to the signal^17^. In regions of low flow rates the noise is totally random and uncorrelated to the signal which makes the noise in those regions more critical to handle^18^. The incidental fluctuations in phase arising from effects other than flow like field inhomogeneity also contribute to noise^19^. Noise arising out of other sources like thermal noise arising from the body^20^ or electrical noise^21^ from the MRI circuit also makes the data corrupted.

Because of such noises, vortices inside the cardiac chambers can only be seen in particular phases of the cardiac cycle predominantly in the ventricular diastole phase as shown in Fig. 1. In some of the phases, the noise may actually hide vortical structures that are present in the data. Consequently, phase Contrast MRI data requires proper denoising operation to ensure proper visual detection and mathematical operations on the vortical structures present inside the data.

Traditionally quite a number of different filters have been developed for denoising purposes. The classic widely popular Median filter is a nonlinear low pass filter which is effective in removing random noise like salt and pepper noise from the data^22^. Wiener filter is an adaptive low pass filter which restores corrupted data by minimizing the mean square error between the pristine and reconstructed data^23^. Gaussian filter is a low pass filter that denoises data by replacing each data point with the weighted average of the neighboring data points^24^. Total variation filter is an edge preserving denoising filter that is based on the principle that the integral of the gradient magnitude of a noisy image is large^25^. For all the common categories of traditional filters, the concept of kernel captures the scale of underlying phenomenology. The question which arises from here is the optimum kernel size to be used for the filtering operation.

Optimal choice of kernel size is necessary to capture underlying phenomenology happening at appropriate scales. Smaller kernel size captures a higher level of variability providing abundant information while larger kernel size adds stability and provides global features of the underlying system. Large kernels may add to smoothing effect and small kernels preserve granularity.

This paper presents a method to determine the optimum kernel size based on some synthetic vortex dominated flow fields generated from harmonic sinusoidal functions with added Gaussian noise. The approach has been applied to patient data obtained using MRI imaging. Its ability to reveal cardiac vortices in flow-fields with low signal-to-noise ratios and its utility in improving intuitive visualization of vortices is presented.

## Acquisition of human cardiac flow data

High temporal resolution phase contrast MRI pulse sequence was used to obtain the velocity data. The MRI scans were done in a 1.5 T SIEMENS Scanner. The temporal resolution of the pulse sequence was 14 ms, the imaging matrix was 192 × 192 with spatial resolution 1.5 × 1.5 × 8 mm^3^ and echo train length 3. The VENC parameter was 90 cm/s and receiver bandwidth 500 Hz/pixel. GRAPPA acceleration scheme was used to limit the acquisition within 20 s per frame.

The acquisition was performed on 8 young healthy volunteers having no known history of cardiac illness with age below 30 years. The data was obtained during breath holds of duration 15 s. Three 4 chamber long axis slices were obtained. 18 long axis cine images were obtained separately throughout the cardiac cycle. This study is a gender neutral study.

The study protocol was approved by the Institutional Review Board of Yale School of Medicine (HIC No.: 0712003302). All methods involved in the study were carried out in accordance with guidelines and regulations of Yale School of Medicine. Written informed consent was obtained from all participating subjects.

The velocity contour of Fig. 1 is plotted from the PC MRI data thus obtained.

### Ethics approval and consent to participate

Written informed consent was obtained from all participating subjects.

## Optimum Kernel size determination procedure

In this work synthetic two dimensional vortex dominated flow fields have been formulated using sinusoidal harmonic functions comprising of various combinations of amplitudes (A_n_ and B_n_), wavenumbers (ω_n_) and phase shifts (ϕ_n_) as described in the subsequent equations.

1a $$u = \sum_{n =1}^{K}\left({A}_{n}cos\left({\omega }_{n}y+{\varnothing }_{n}\right)+{B}_{n}sin\left({\omega }_{n}y+{\varnothing }_{n}\right)\right)$$

1b $$v = \sum_{n =1}^{K}\left({A}_{n}cos\left({\omega }_{n}x+{\mathrm{\varnothing }}_{n}\right)+{B}_{n}sin\left({\omega }_{n}x+{\mathrm{\varnothing }}_{n}\right)\right) $$

For simplicity, the value of K is chosen in the range from 1 to 3. These flow fields are rotational and satisfy the continuity equation.

**Figure 2:**
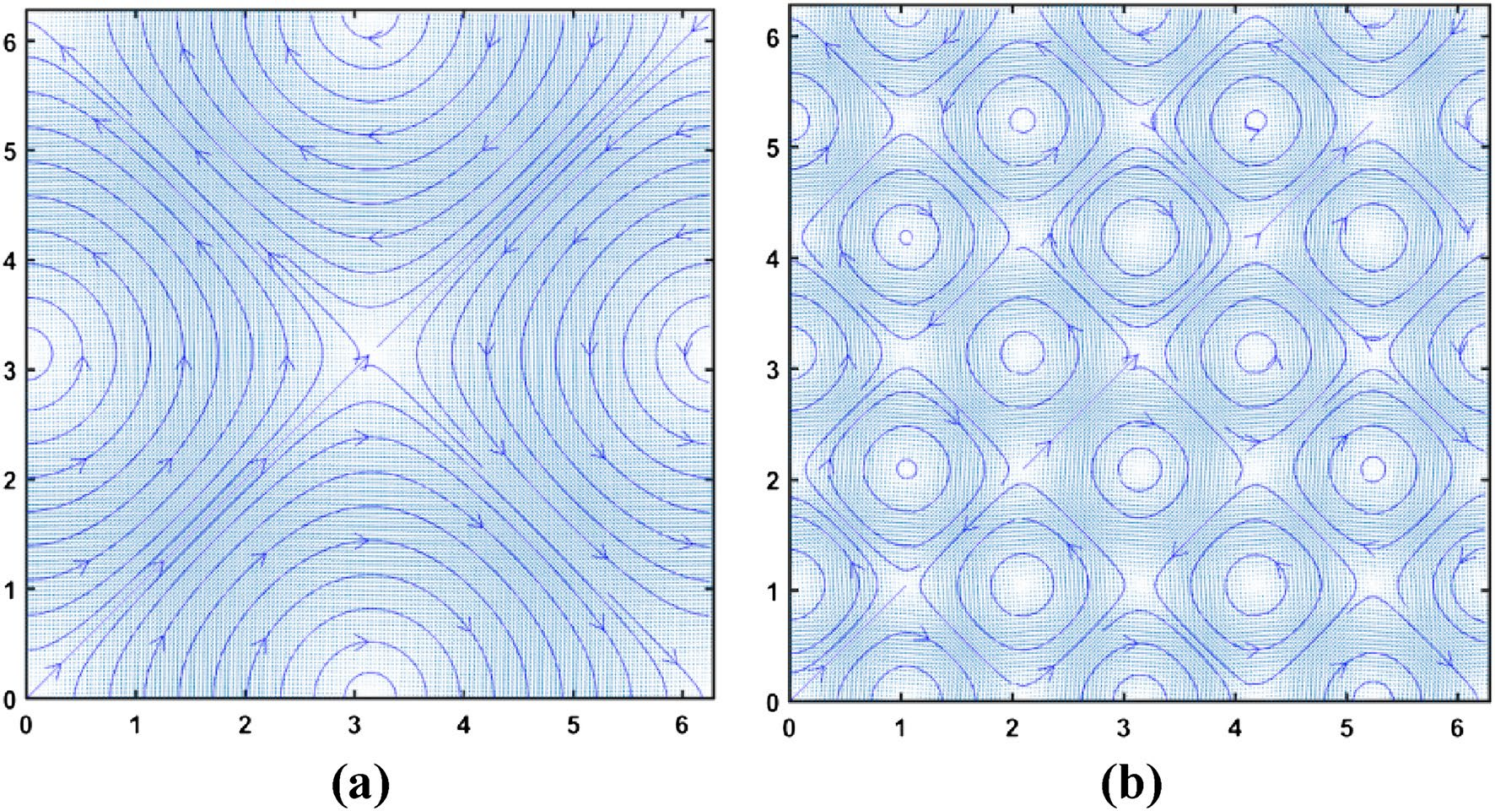
Formulation of vortex dominated flow fields using unimodal harmonic sinusoidal function of (**a**) lower wavenumber, (**b**) higher wavenumber.

Two different flow fields formulated by the above equations are shown in Fig. 2. The flow field of Fig. 2a has been formulated as per the following equations.

$$ \begin{gathered} {\text{u }} = {\text{ sin}}\left( {\text{y}} \right) \hfill \\ {\text{v }} = {\text{ sin}}\left( {\text{x}} \right) \hfill \\ \end{gathered} $$

while the flow field of Fig. 2b has been formulated using the following equations.

$$ \begin{gathered} {\text{u }} = {\text{ sin}}\left( {{\text{3y}}} \right) \hfill \\ {\text{v }} = {\text{ sin}}\left( {{\text{3x}}} \right) \hfill \\ \end{gathered} $$

The value of x and y in both cases range from 0 to 2π.

**Figure 3:**
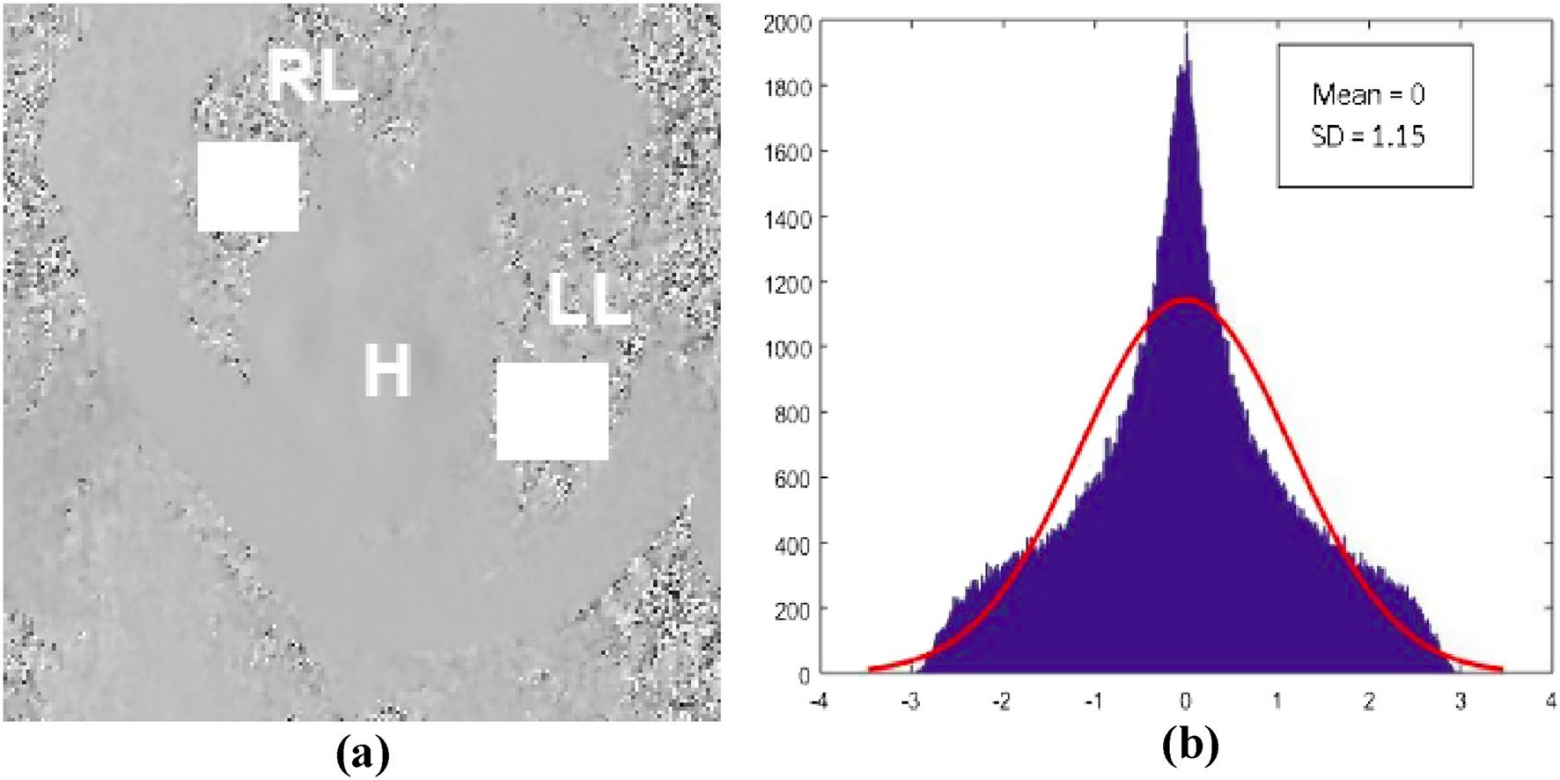
Determination of nature of noise present in PC MRI Data (**a**) Segmentation of a rectangular region from two lungs. (**b**) Fitting of a normal distribution to the histogram of the segmented pixel values. RL represents the location of the right lung, LL represents the location of the left lung, H represents the location of the heart.

Gaussian noise of certain amplitude/Standard Deviation (SD) is added to the formulated harmonic sinusoidal velocity fields. The magnitude of noise to be added to the flow fields is benchmarked with the noise in the lung region of the PC MRI Data (Fig. 3). A rectangular region is segmented from both the lungs in the PC MRI phase data (Fig. 3a), then a normal distribution is fitted to the histogram of the segmented pixel values (Fig. 3b) and the corresponding parameters, i.e., the mean and the Standard Deviation (SD) are obtained.

Vorticity field is calculated from the velocity fields defined in Eqs. (1a, 1b) by the formulation described in Eq. (2).

2 $${\omega }_{ij }= \frac{1}{{N}^{2}}\sum_{m=1}^{N}\sum_{n=1}^{N}\left(\frac{v\left(i+m,j+n\right)-v\left(i-m,j+n\right)}{2m\Delta x}-\frac{u\left(i+m,j+n\right)-u\left(i+m,j-n\right)}{2n\Delta y}\right)$$

where the vorticity is averaged over an interrogation window of size N. Physically vorticity is a measure of the local rotation of the fluid element around the point (i, j).

**Figure 4:**
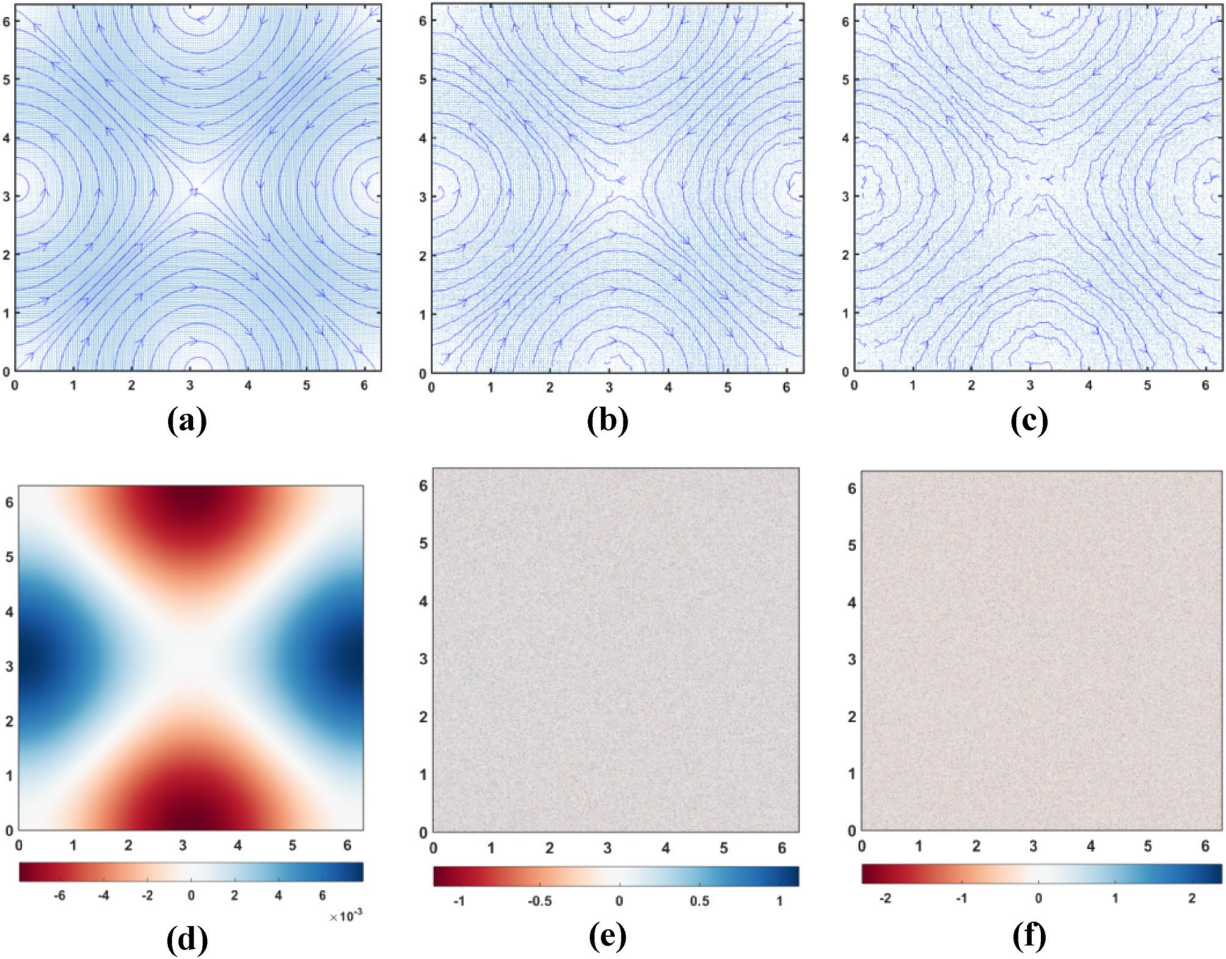
(**a**) Pristine velocity field. (**b**) Velocity field with added Gaussian noise of SD = 0.2. (**c**) Velocity field with added Gaussian noise of SD = 0.4. (**d**) Pristine vorticity field. (**e**) Vorticity field with added Gaussian noise of SD = 0.2. (**f**) Vorticity field with added Gaussian noise of SD = 0.4.

It is evident from Fig. 4 that the additive Gaussian noise deforms the vorticity fields manifold times more than the velocity fields. This is due to the fact that vorticity as per its definition represents frequency of spatial change in velocity. Figure 4a shows the pristine velocity field while Fig. 4b and Fig. 4c shows the velocity fields corrupted with Gaussian noise of SD = 0.2 and SD = 0.4 respectively. Figure 4d shows the pristine vorticity field while Fig. 4e,f shows the vorticity fields corrupted with Gaussian noise of SD = 0.2 and SD = 0.4 respectively.

The corrupted velocity fields are filtered with Median filter of gradually increasing kernel sizes and the deviation, i.e., root mean square error (RMSE) between the pristine and filtered vorticity fields are plotted as a function of the kernel size.

The RMSE is defined by Eq. (3) as

3 $$RMSE= \sqrt{\stackrel{-}{\left(\omega -{\upomega }^{f}\right).\left(\omega -{\omega }^{f}\right)}}$$

where ω is the vorticity field calculated from the pristine velocity field and ω^f^ is the vorticity field calculated from the restored velocity field according to the formulation given in Eq. (2).

**Figure 5:**
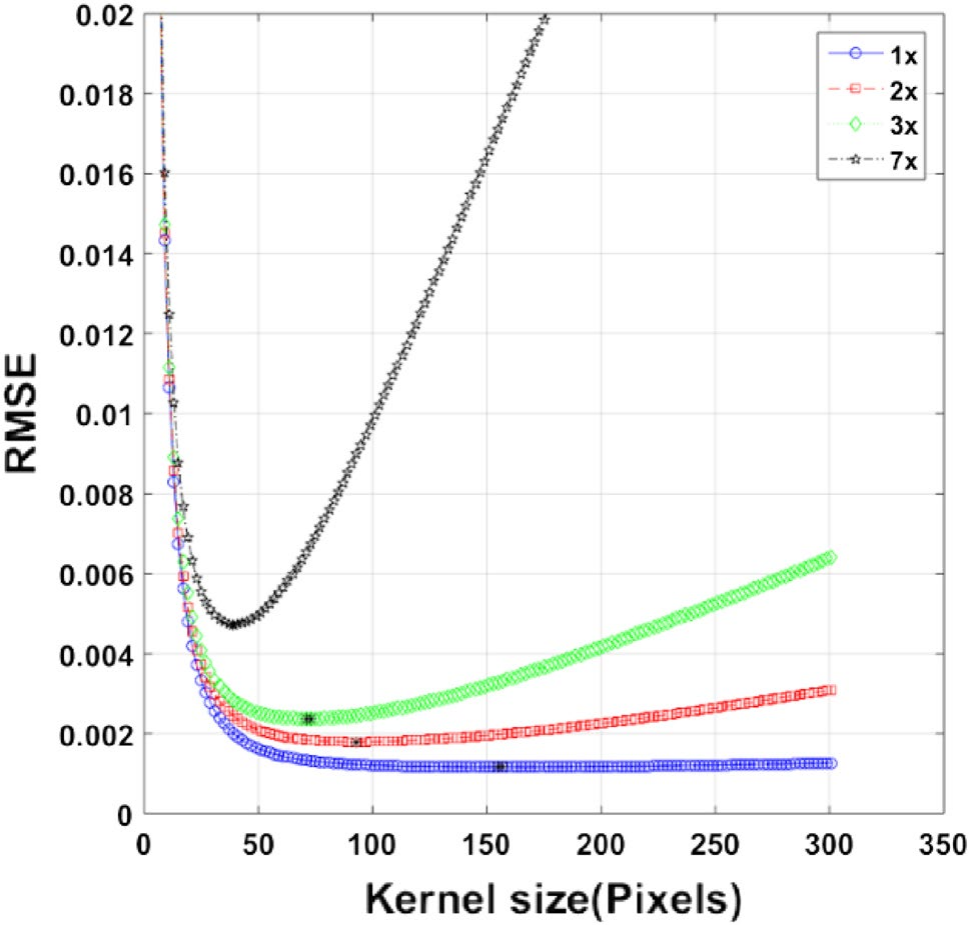
Existence of a sweet spot (i.e., a kernel size corresponding to minimum RMSE) for a given flow field. Y axis represents the RMSE (root mean square error) between the pristine and restored vorticity field. The points marked by black asterisk on each curve correspond to the sweet spot.

Figure 5 shows the RMSE vs. kernel size plot for four different unimodal velocity fields of increasing wavenumbers. It is observed that with the increase in wavenumber and consequent decrease in wavelength the minima of the RMSE vs. kernel size plot shifts towards the left. This minima is the optimal value of the kernel size to be used for filtering the flow field. From this it can be inferred that the optimal kernel size for filtering a velocity field roughly scales as a fraction of the underlying wavelength present in the field.

In Fig. 5, when the increment in the kernel size is small enough, the RMSE vs. kernel size curve collapses up to a certain kernel size due to dominance of noise over signal in lower kernel size regime. As the kernel size crosses a certain value, the curves get separated. The value of this separation kernel is unique to each mode or wavenumber. From this separation point, the signal starts to dominate over noise. As the kernel size augments further, the curves reach to a minimum value of RMSE at a certain kernel size (i.e., the sweet spot) beyond which the RMSE starts to rerise. The sweet spot is the kernel size at which the filter performs optimally. On taking a kernel size beyond this optimal point, the statistical sample size increases to a limit which induces heightened non-uniformity, thereby surging the RMSE.

For the purpose of vortex size determination, a method which uses a Galilean invariant version of Normalized Angular Momentum (NAM) is used^26^.

The method identifies point P as a vortex center if $\left|{\Gamma }_{1}(P)\right|$ defined by Eq. (4) ranges from 0.9 to 1 at that point.

4 $${\Gamma }_{1}\left(P\right)= \frac{1}{S}\int \frac{\left(PM\Lambda {U}_{M}\right).z}{\Vert PM\Vert .\Vert {U}_{M}\Vert }dS= \frac{1}{S}\int sin\left({\Theta }_{M}\right)dS$$

where M lines in S and S is a two dimensional area surrounding P. z is the unit vector normal to the measurement plane. ${\theta }_{M}$ is the angle between the velocity vector ${U}_{M}$ and radius vector PM.

The boundaries of the vortices are determined based on some threshold value of the NAM. The vortex size for the flow field shown in Fig. 2b is obtained by this method and the corresponding optimal kernel size is obtained from the minima of the 3 × curve in Fig. 5.

The vortex size for the human cardiac flow field shown in Fig. 1 is obtained by the same method described in^26^. By equating a ratio of kernel size to the vortex size between the cardiac and synthetic flow field, the optimal kernel size corresponding to the cardiac vortex size is obtained. The value of the optimal kernel size corresponding to the cardiac vortex size is approximately 13.

**Figure 6:**
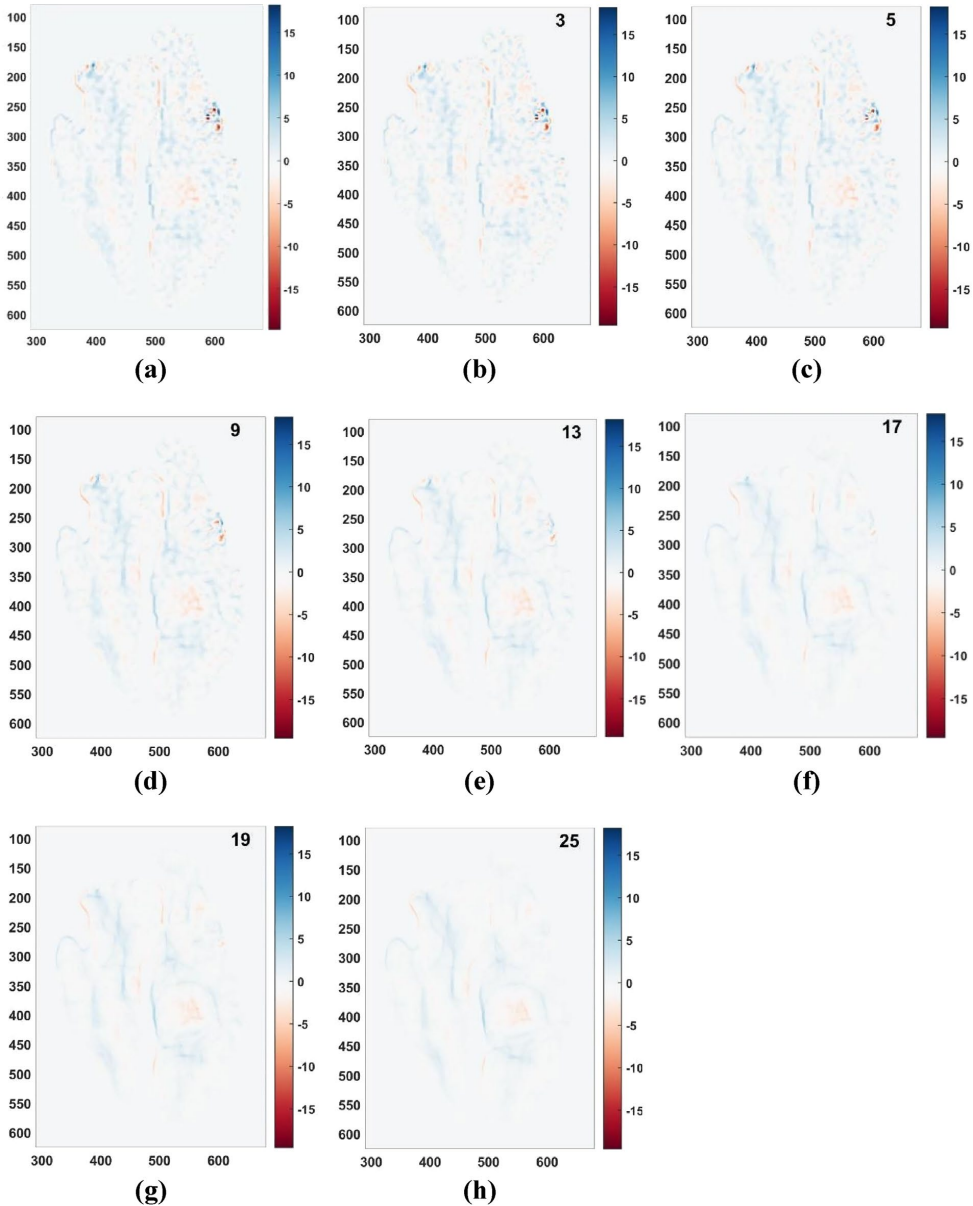
(**a**) Raw vorticity field directly obtained from the cardiac flow field shown in Fig. 1. Vorticity fields obtained after filtering the cardiac flow field shown in Fig. 1 using kernel of size (**b**) 3 × 3 (**c**) 5 × 5 (**d**) 9 × 9 (**e**) 13 × 13 (**f**) 17 × 17 (**g**) 19 × 19 (h) 25 × 25. X and Y axes are spatial coordinates having units of pixels. Unit of colorbar, i.e., vorticity is 1/s.

The cardiac flow field shown in Fig. 1 is filtered by this optimal kernel size value of 13 and also with some values preceding and succeeding this value (Fig. 6). This is done to demonstrate that the filtered cardiac flow fields behave roughly in a similar fashion to the behavior of the synthetic flow fields which is shown in Fig. 5.

It is evident from Fig. 6 that at kernel sizes near to the optimal value of 13, the vorticity fields are more physically intuitive with the edges of the boundary layer and vortical boundaries getting more clarity. At values much lower than the optimal, the noise has much dominance over the signal while at values much higher than the optimal the signal has lost its clarity.

**Figure 7:**
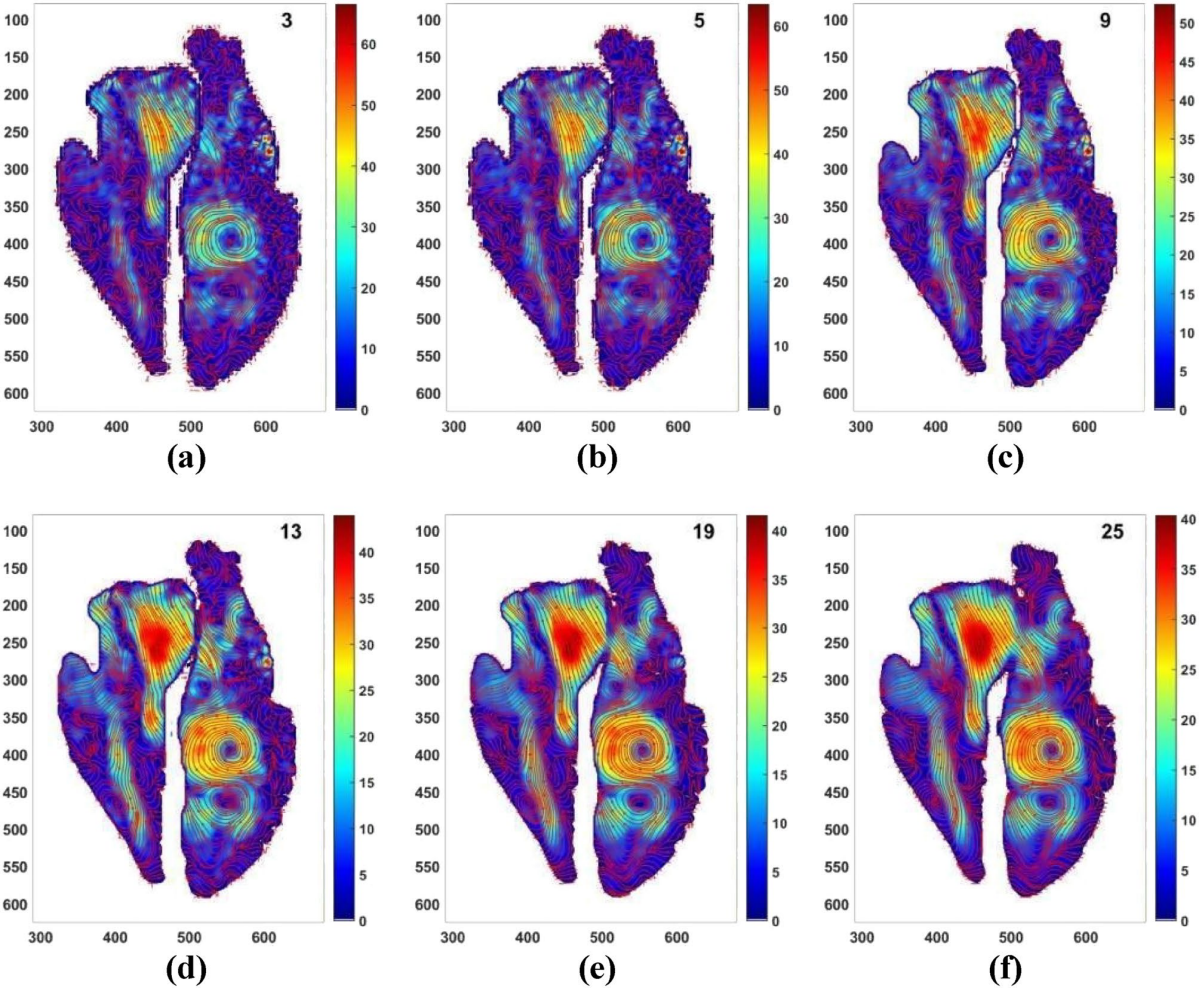
Velocity fields obtained after filtering the cardiac flow field shown in Fig. 1 using kernel of size (**a**) 3 × 3 (**b**) 5 × 5 (**c**) 9 × 9 (**d**) 13 × 13 (**e**) 19 × 19 (**f**) 25 × 25. X and Y axes are spatial coordinates having units of pixels. Unit of colorbar, i.e., velocity is cm/s.

Figure 7 shows the velocity fields corresponding to the vorticity fields of Fig. 6. It is observed that at kernel sizes near to the optimal value of 13, the features of the flow field which were not visible in the original image (Fig. 1) have become conspicuous. The strong velocity gradients at the vortical region inside the left ventricle and also a strong gradient of velocity inside the right atrium have become very prominent.

## Summary and conclusions

In this work, an adaptive kernel size based flow field recovery process from Phase Contrast MRI Data has been presented which makes use of median filter to restore the noise corrupted flow fields with kernel size derived by scaling the ratio of kernel size to vortex size with some synthetic harmonic flow fields.

Synthetic vortex dominated flow fields made up of harmonic sinusoidal functions were generated combining different amplitudes, wave numbers, phases which were spoiled with additive Gaussian noise and subsequently restored by median filtering with increasing kernel sizes. It was observed that the addition of Gaussian noise distorts the vorticity field manifold times more than the velocity field. Sensitivity of the deviation of the restored vorticity field from its pristine version to the kernel size was studied.

Cardiac flow field obtained through Phase Contrast MRI was median filtered using the optimal kernel size obtained from the technique described in this paper as well as some kernel sizes preceding and succeeding that value. It was observed that filtering each image with an adaptive kernel size which is scaled with the vortex size generates a much better resolution vorticity field in comparison with the other kernel sizes. This advocates that the technique described in this paper will be instrumental in better study of vortex and vorticity related parameters from velocity data obtained through PC MRI. Those parameters can eventually be established as biomarkers for various cardiac pathologies that can be used by radiologists and cardiologists for better pathology detection, diagnosis, prognosis prediction and therapeutic intervention. The better resolution of the boundary layer vorticity in the adaptively filtered image can also facilitate retrospective myocardial wall boundary detection and valve leaflet tracking.

## Data Availability

Data is available on request from the corresponding author.

## References

[1] Arvidsson PM, Kovács SJ, Töger J, Borgquist R, Heiberg E, Carlsson M, Arheden H (2016). Vortex ring behavior provides the epigenetic blueprint for the human heart. Sci. Rep..

[2] Pedrizzetti G, La Canna G, Alfieri O, Tonti G (2014). The vortex—an early predictor of cardiovascular outcome?. Nat. Rev. Cardiol..

[3] Reiter G, Reiter U, Kovacs G, Kainz B, Schmidt K, Maier R, Olschewski H, Rienmueller R (2008). Magnetic resonance–derived 3-dimensional blood flow patterns in the main pulmonary artery as a marker of pulmonary hypertension and a measure of elevated mean pulmonary arterial pressure. Circ. Cardiovasc. Imaging.

[4] Reiter G, Reiter U, Kovacs G, Olschewski H, Fuchsjäger M (2015). Blood flow vortices along the main pulmonary artery measured with MR imaging for diagnosis of pulmonary hypertension. Radiology.

[5] Gharib M, Rambod E, Kheradvar A, Sahn DJ, Dabiri JO (2006). Optimal vortex formation as an index of cardiac health. Proc. Natl. Acad. Sci..

[6] Kheradvar R, Assadi A, Falahatpisheh PP (2012). Assessment of transmitral vortex formation in patients with diastolic dysfunction. J. Am. Soc. Echocardiogr..

[7] Garcia J, Sheitt H, Bristow MS, Lydell C, Howarth AG, Heydari B, Prato FS, Drangova M, Thornhill RE, Nery P, Wilton SB (2020). Left atrial vortex size and velocity distributions by 4D flow MRI in patients with paroxysmal atrial fibrillation: Associations with age and CHA2DS2-VASc risk score. J. Magn. Reson. Imaging.

[8] Hong GR, Pedrizzetti G, Tonti G, Li P, Wei Z, Kim JK, Baweja A, Liu S, Chung N, Houle H, Narula J (2008). Characterization and quantification of vortex flow in the human left ventricle by contrast echocardiography using vector particle image velocimetry. JACC Cardiovasc. Imaging.

[9] Kanski M, Arvidsson PM, Töger J, Borgquist R, Heiberg E, Carlsson M, Arheden H (2015). Left ventricular fluid kinetic energy time curves in heart failure from cardiovascular magnetic resonance 4D flow data. J. Cardiovasc. Magn. Reson..

[10] Querzoli G, Fortini S, Cenedese A (2010). Effect of the prosthetic mitral valve on vortex dynamics and turbulence of the left ventricular flow. Phys. Fluids.

[11] Domenichini F, Querzoli G, Cenedese A, Pedrizzetti G (2007). Combined experimental and numerical analysis of the flow structure into the left ventricle. J. Biomech..

[12] Loke YH, Capuano F, Kollar S, Cibis M, Kitslaar P, Balaras E, Reiber JH, Pedrizzetti G, Oliveri L (2022). Abnormal diastolic hemodynamic forces: A link between right ventricular wall motion, intracardiac flow, and pulmonary regurgitation in repaired tetralogy of fallot. Front. Cardiovasc. Med..

[13] Franco P, Sotelo J, Guala A, Dux-Santoy L, Evangelista A, Rodriguez-Palomares J, Mery D, Salas R, Uribe S (2022). Identification of hemodynamic biomarkers for bicuspid aortic valve induced aortic dilatation using machine learning. Comput. Biol. Med..

[14] Edelstein WA, Bottomley PA, Pfeifer LM (1984). A signal-to-noise calibration procedure for NMR imaging systems. Med. Phys..

[15] Song SM, Napel S, Glover GH, Pelc NJ (1993). Noise reduction in three-dimensional phase-contrast MR velocity measurements. J. Magn. Reson. Imaging.

[16] Dumoulin CL, Souza SP, Darrow RD, Pelc NJ, Adams WJ, Ash SA (1991). Simultaneous acquisition of phase-contrast angiograms and stationary-tissue images with Hadamard encoding of flow-induced phase shifts. J. Magn. Reson. Imaging.

[17] Henriques, R. N., Ianus, A., Novello, L., Jovicich, J., Jespersen, S., & Shemesh, N. Efficient PCA denoising of spatially correlated MRI data. *bioRxiv* 2023-03 (2023). 10.1162/imag_a_00049 PMC12180759 40799709

[18] Conturo TE, Smith GD (1990). Signal-to-noise in phase angle reconstruction: Dynamic range extension using phase reference offsets. Magn. Reson. Med..

[19] Andersen AH, Kirsch JE (1996). Analysis of noise in phase contrast MR imaging. Med. Phys..

[20] Rarrazaval P, Firoozabadi AD, Uribe S, Tejos C, Sing-Long C (2019). Noise estimation for the velocity in MRI phase-contrast. Magn. Reson. Imaging.

[21] Wymer DT, Patel KP, Burke WF, Bhatia VK (2020). Phase-contrast MRI: Physics, techniques, and clinical applications. Radiographics.

[22] Chan RH, Ho CW, Nikolova M (2005). Salt-and-pepper noise removal by median-type noise detectors and detail-preserving regularization. IEEE Trans. Image Process..

[23] Saluja, R., & Boyat, A. Wavelet based image denoising using weighted high pass filtering coefficients and adaptive wiener filter. In *2015 International Conference on Computer, Communication and Control (IC4)*, 1–6 (2015).

[24] Makandar BH (2015). Image enhancement techniques using highpass and lowpass filters. Int. J. Comput. Appl..

[25] Yu H, Jia S, Liu Y, Dong J (2020). Phase coherent noise reduction in digital holographic microscopy based on adaptive total variation. Opt. Lasers Eng..

[26] Graftieaux L, Michard M, Grosjean N (2001). Combining PIV, POD and vortex identification algorithms for the study of unsteady turbulent swirling flows. Meas. Sci. Technol..

